# Giant splenic abscess caused by *Salmonella enterica*

**DOI:** 10.1002/ccr3.4003

**Published:** 2021-03-19

**Authors:** Francesk Mulita, Elias Liolis, Levan Tchabashvili, Fotios Seretis, Fotios Iliopoulos, Nikolas Drakos, Ioannis Maroulis, Michail Vailas

**Affiliations:** ^1^ Department of General Surgery General University Hospital of Patras Achaia Greece; ^2^ Department of Internal Medicine General University Hospital of Patras Achaia Greece

**Keywords:** percutaneous drainage, *Salmonella enterica*, splenectomy, splenic abscess

## Abstract

Splenectomy is the gold standard for treating a splenic abscess, when percutaneous drainage fails or is less likely to be successful.

## Graphical abstract

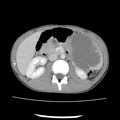

## 1 CASE DESCRIPTION

**FIGURE 1:**
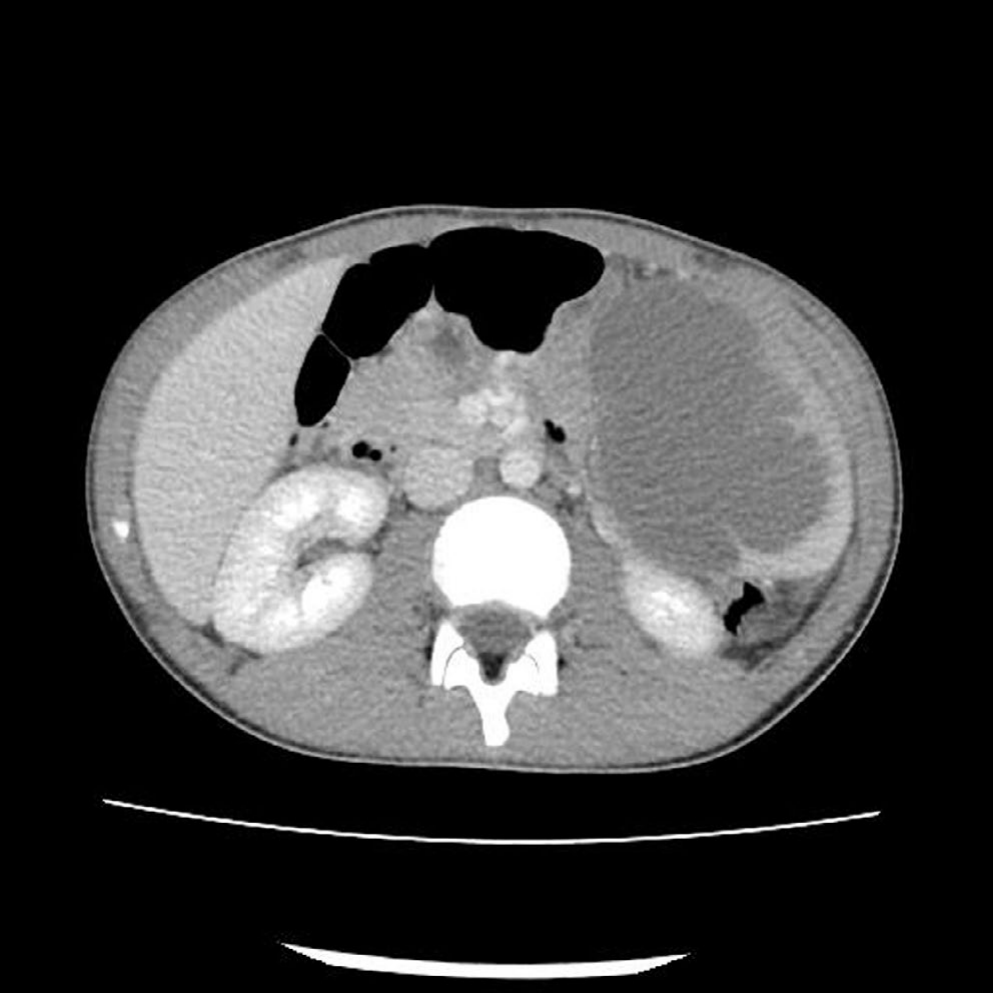
Abdominal CT scan of a 14‐year‐old boy. The spleen contained a large single abscess of 13 cm × 9 cm

A 14‐year‐old boy with free medical history and no surgical intervention was admitted to our hospital with high‐grade fever and abdominal pain lasting for 4 days. On examination, the patient's temperature was 39, heart rate was 105 beats per minute, blood pressure was 110/70 and respiratory rate was 12 breaths per minute. His abdomen was soft, without distension, and only mild tenderness in the upper left quadrant was found. The initial white blood cells were 11.77 K/μL, and C‐reactive protein level was 20.8 U/L. Liver and renal function test and serum amylase were normal. A computed tomography (CT) of the abdomen showed a large abscess of the spleen, of an average size of 13 cm × 9 cm (Figure 1).

Empirical treatment with meropenem and vancomycin was started, and percutaneous aspiration of the lesion was performed under CT guidance. The material was sent for culture, which led to the development of *Salmonella enterica*. Splenectomy was performed 38 days after percutaneous aspiration because of abscess's considerable size. The operation was completed successfully. The spleen weighed 362 g. Histopathology revealed a giant splenic epidermoid cyst with a wall consisting of fibrous tissue and accumulation of necrotic tissue.

Abscess of the spleen is a very rare clinical problem and carries very high mortality reaching more than 70%, if the diagnosis is missed.^1^ According to the literature, treatment of splenic abscess includes conservative measures as well as surgical intervention. In children and in cases of solitary abscesses with a thick wall, percutaneous aspiration may be performed. Otherwise, splenectomy is the preferred approach in most cases. However, treatment should be customized for each patient.^2^

## CONFLICT OF INTEREST

There are no conflicts of interest to declare.

## AUTHOR CONTRIBUTIONS

FM, EL, LT, FS, FI and ND: contributed to the clinical data collection and prepared the case report. FM, IM and MV: contributed to the design of the case report presentation and performed the final revision of the manuscript.

## ETHICAL APPROVAL

This report for a clinical image was conducted in accordance with the Declaration of Helsinki. The collection and evaluation of all protected patient health information was performed in a Health Insurance Portability and Accountability (HIPAA) complaint manner. A formal informed consent was obtained from the patient prior to the publication of this article.

## Data Availability

Data available on request from the authors.
